# Supplementary material for: Standardised 25-Step Traditional Thai Massage (TTM) Protocol for Treating Office Syndrome (OS)
Source: Int J Environ Res Public Health. 2023 Jun 16;20(12):6159. doi: 10.3390/ijerph20126159 (PMC10297894; doi:10.3390/ijerph20126159)
Supplement: Supplementary file 1 [file ijerph-20-06159-s001.zip › ijerph-2288455-supplementary.pdf]

Supplementary A: Pattern of massage techniques

**Force**	**Light**	**Moderate**		**Firm**	
**Area of contact (Thumb)**	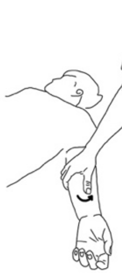	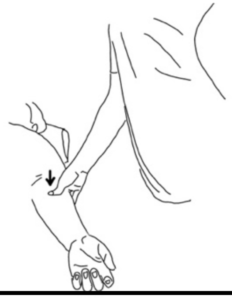	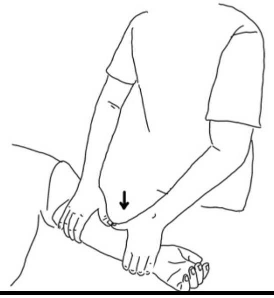	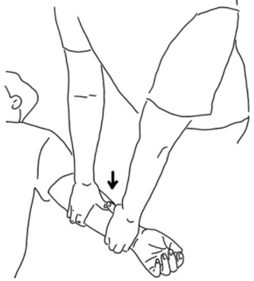	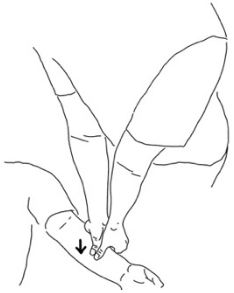
**Massage Technique**	Thumb Circle (TC)	Thumb Press (TP)	Double Thumb press (DTP)	Cross Thumb Press (CTP)	Side Thumb Press (STP)
**Force**	**Light**	**Moderate**		**Firm**	
**Area of contact (Palm and Hell of hand)**	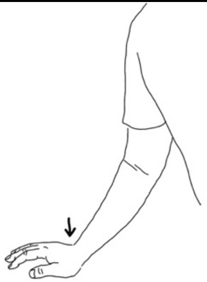	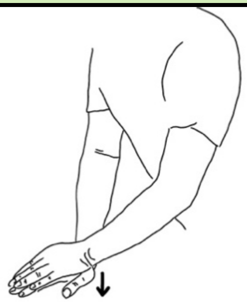	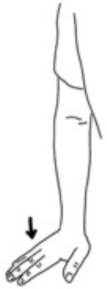	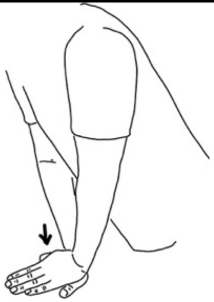	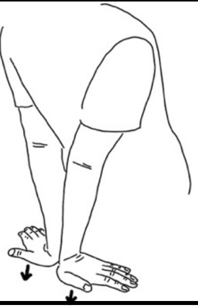
**Massage Technique**	Heel Press (HP)	Double Heel Press (DHP)	Palm Press (PP)	Double Palm Press (DPP)	Side Palm Press (SPP)

According to Standardized 25 step TTM protocol: Force was categorized by the therapists' body weight into three levels as light (no body weight), moderate (body weight with bend elbow), and firm (body weight with straight elbow); Area of contact contained thumb, palm, and heel of hand.

Supplementary B: The standardized 25 steps of the TTM protocol

The standardized 25 steps of the TTM protocol for Office Syndrome including 25 discrete steps over 90 minutes of whole-body massage.

<table>
<tr><th colspan="2">25-steps of the TTM</th><th>Path</th><th colspan="2">Example</th></tr>
<tr><th colspan="5">Body position I: Supine lying</th></tr>
<tr><td colspan="2">1. Dorsal Foot</td><td rowspan="3">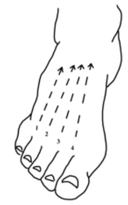</td><td colspan="2" rowspan="3">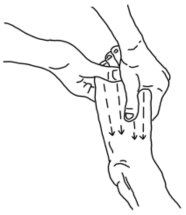</td></tr>
<tr><td>Timing (min): (time)</td><td>01.30: (1)</td></tr>
<tr><td>Massage Technique</td><td>TP</td></tr>
<tr><td colspan="2">2. Anterior Leg and Thigh</td><td rowspan="3">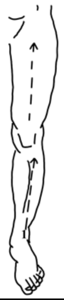</td><td rowspan="3">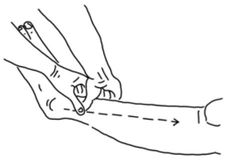</td><td rowspan="3">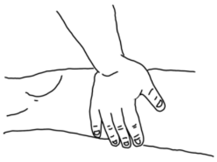</td></tr>
<tr><td>Timing (min): (time)</td><td>01.30: (1)</td></tr>
<tr><td>Massage Technique</td><td>CTP, HP</td></tr>
<tr><td colspan="2">3. Lateral Thigh</td><td rowspan="3">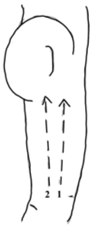</td><td colspan="2" rowspan="3">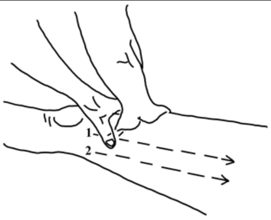</td></tr>
<tr><td>Timing (min): (time)</td><td>01.00: (1)</td></tr>
<tr><td>Massage Technique</td><td>CTP</td></tr>
<tr><td colspan="2">4. Femoral Artery Occlusion</td><td rowspan="3">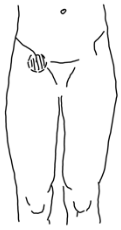</td><td colspan="2" rowspan="3">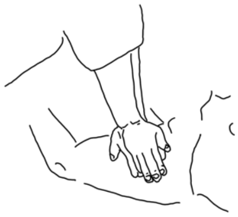</td></tr>
<tr><td>Timing (min): (time)</td><td>00.45: (1)</td></tr>
<tr><td rowspan="2">Massage Technique</td><td>DPP</td></tr>
<tr><td>Suggestion</td><td colspan="3">The hip should be a slight external rotation.</td></tr>
<tr><td colspan="2">5. Palmar Hand</td><td rowspan="3">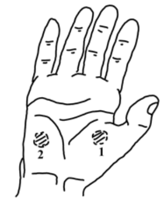</td><td colspan="2" rowspan="3">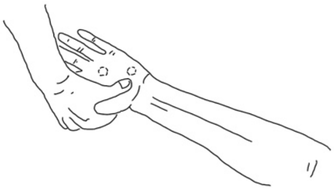</td></tr>
<tr><td>Timing (min): (time)</td><td>00.45: (1)</td></tr>
<tr><td>Massage Technique</td><td>TP</td></tr>
<tr><td colspan="2">6. Anterior Arm</td><td rowspan="3">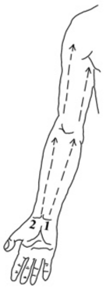</td><td colspan="2" rowspan="3">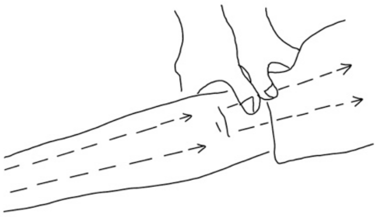</td></tr>
<tr><td>Timing (min): (time)</td><td>01.30: (1)</td></tr>
<tr><td>Massage Technique</td><td>ante-arm: STP
arm: DTP, CTP</td></tr>
</table>

25-steps of the TTM		Path	Example
7. Dorsal Hand		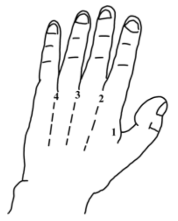	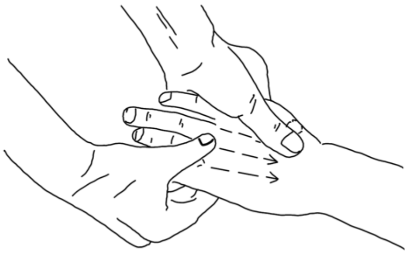
Timing<br>(min): (time)	01.00: (1)		
Massage Technique	TP		
8. Posterior Arm		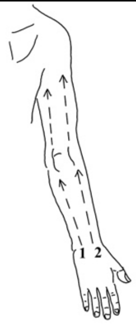	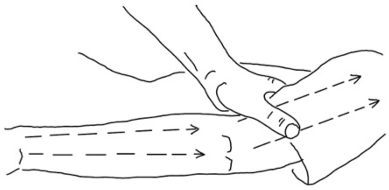
Timing<br>(min): (time)	01.30: (1)		
Massage Technique	TP, DTP, CTP		
	Suggestion	Lifting the arm should be done to give massage at the posterior ante arm.	
9. Anterior shoulder		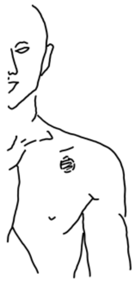	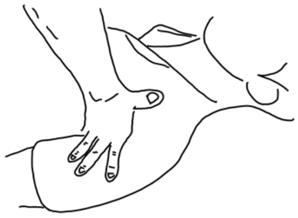
Timing<br>(min): (time)	00.20: (1)		
Massage Technique	TP, TC		
10. Brachial Artery Occlusion		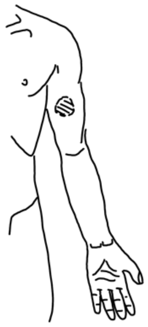	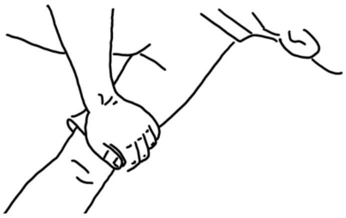
Timing<br>(min): (time)	00.45: (1)		
Massage Technique	PP, HP		
	Suggestion	The shoulder should be abduction at 90 degrees, and applying force should be located at mid-arm.	
**Repeated the right side with same steps (1-10)			
**Body position II: Side lying**			
11. Plantar Foot		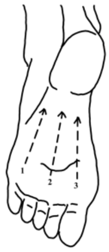	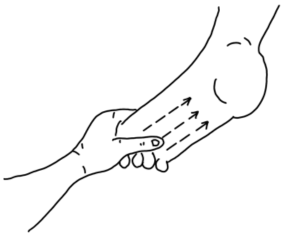
Timing<br>(min): (time)	01.30: (1)		
Massage Technique	TP		

25-steps of the TTM		Path	Example
12. Medial Leg & Thigh		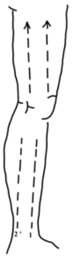	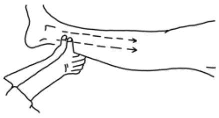 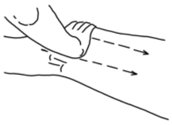
Timing<br>(min): (time)	01.30: (1)		
Massage Technique	Leg; STP<br>Thigh; PP		
13. Lateral Leg & Thigh		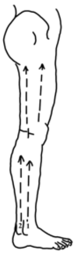	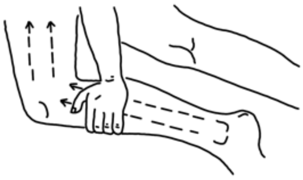
Timing<br>(min): (time)	02.00: (1)		
Massage Technique	PP		
14. Gluteus		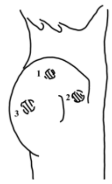	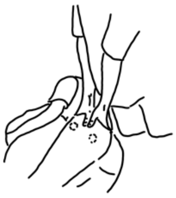
Timing<br>(min): (time)	00.45: (1)		
Massage Technique	STP		
15. Lateral Lower & Upper back		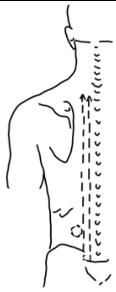	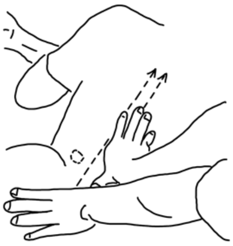
Timing<br>(min): (time)	05.00: (2)		
Massage Technique	Path 1: DTP<br>Path 2: CTP		
	Suggestion	Path 1, 2 and Point are the distance away from the vertebral spine at one, two, and three finger's therapist, respectively.	
16. Scapular		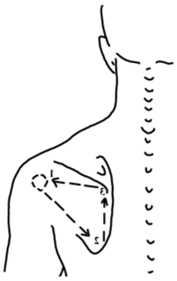	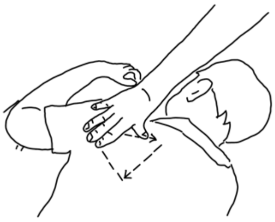
Timing<br>(min): (time)	02.00: (2)		
Massage Technique	TP		
17. Posterior Shoulder & Neck		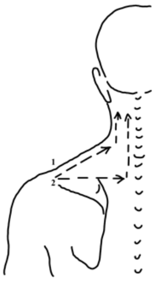	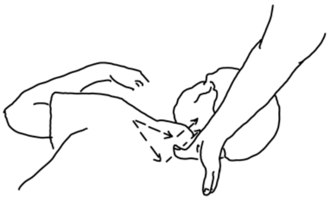
Timing<br>(min): (time)	02.00: (2)		
Massage Technique	TP, STP		

25-steps of the TTM		Path	Example
18. Lateral Shoulder & Head		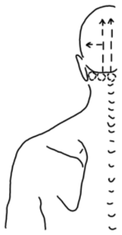	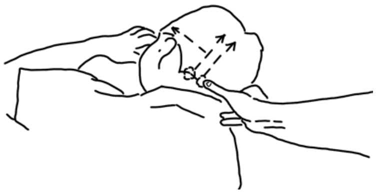
Timing (min): (time)	02.00: (1)		
Massage Technique	TC		
**Repeated the right side with same steps (11-18)			
**Body position III: Prone on stomach**			
19. Posterior Leg		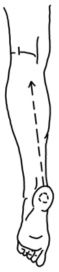	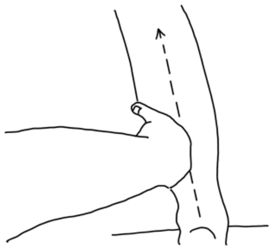
Timing (min): (time)	01.00: (1)		
Massage Technique	HP, PP		
20. Posterior Thigh		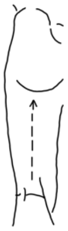	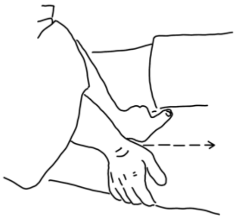
Timing (min): (time)	01.00: (1)		
Massage Technique	SPP		
21. Posterior Lower & Upper back		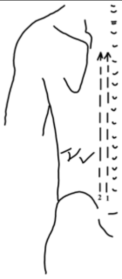	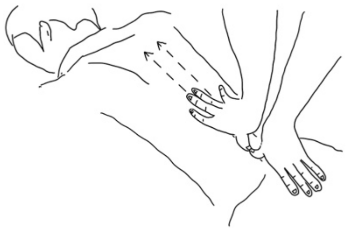
Timing (min): (time)	04.00: (2)		
Massage Technique	Path 1; DTP<br>Path 2; CTP		
	Suggestion	Path one should be applying force at 90 degrees with parallel on the vertebral column and path two should be applying force at 45 degrees with oblique on the vertebral column.	
**Repeated the right side with same steps (19-21)			
**Body position IV: Supine lying**			
22. Head & Face		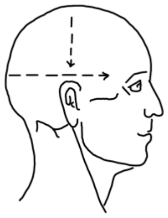	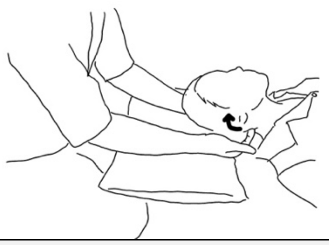
Timing (min): (time)	02.00: (1)		
Massage Technique	TC		
	Suggestion	Point 3 is occipital muscle stretching by four fingers' therapist in both hands.	

25-steps of the TTM		Path	Example	
23. Lower Extremity Stretching		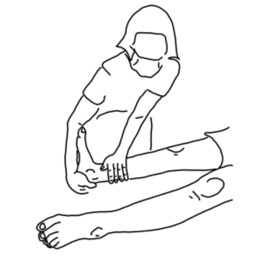	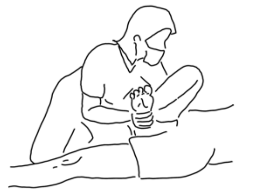	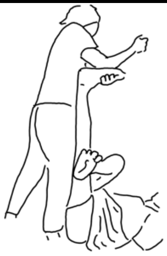
Timing (min): (time)	03.00: (1)	i (ankle & leg)	ii (posterior hip)	iii (posterior thigh & foot)
24. Trunk Stretching		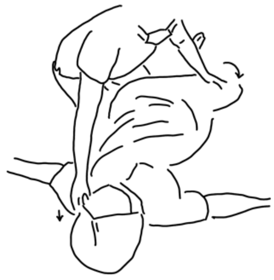	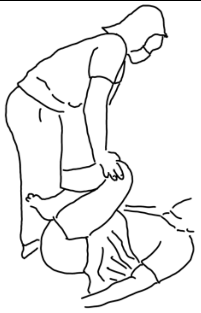	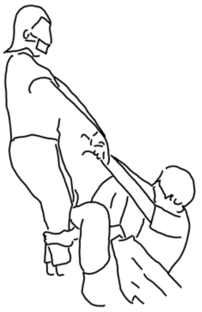
Timing (min): (time)	03.30: (2)	i (trunk)	ii (trunk with hip)	iii (trunk with upper limb pull)
**Body position V: Sitting**				
25. Upper Extremity Stretching		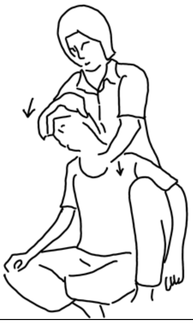	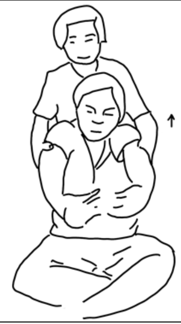	
Timing (min): (time)	01.30: (1)	i (shoulder)	ii (extremity)	
Symbol	◄ - - - Direction to apply force	◄ - - - - - 2 ◄ - - - - - 1 Sequence of massage	Major signal points (MaSP)	
